# Pharmacokinetics of DA-6886, A New 5-HT_4_ Receptor Agonist, in Rats

**DOI:** 10.3390/pharmaceutics14040702

**Published:** 2022-03-25

**Authors:** Dae Young Lee, Hee Eun Kang

**Affiliations:** 1 Research Center, Dong-A ST Co., Ltd., Yongin 17073, Korea; dylee@donga.co.kr; 2 College of Pharmacy and Integrated Research Institute of Pharmaceutical Sciences, The Catholic University of Korea, Bucheon 14662, Korea

**Keywords:** DA-6886, 5-HT_4_ receptor agonist, LC–MS/MS, pharmacokinetics, dose-dependency

## Abstract

DA-6886 is a novel serotonin (5-hydroxytrypamine [5-HT]) receptor 4 agonist for the potential treatment of constipation-predominant irritable bowel syndrome. The purpose of this study was to validate the quantitative assay of DA-6886 in rat plasma and to evaluate the pharmacokinetics and tissue distribution of DA-6886 in rats. The liquid chromatography–tandem mass spectrometry (LC–MS/MS) method for the robust quantification of DA-6886 in rat plasma was successfully validated and applied to the pharmacokinetic studies in rats. The pharmacokinetic parameters of DA-6886 in rats were evaluated following single intravenous or oral administration at three dose levels (2, 10, and 20 mg/kg). DA-6886 exhibited a smaller dose-normalized area under the plasma concentration–time curve (AUC) values and faster clearances in the low-dose group than in the high-dose group following both intravenous and oral administration. The steady-state volume of distribution (*V*_ss_) of DA-6886 was relatively large (4.91–7.84 L/kg), which was consistent with its high distribution to the liver, kidney, lung, and digestive tract, and was dose-independent. After oral administration, the extent of absolute bioavailability (*F*) tended to increase (18.9–55.0%) with an increasing dose. The slope of the log-transformed AUC and/or *C*_max_ values versus log dose was greater than unity and greater for oral administration (~1.9) than for intravenous administration (~1.1). Because the nonlinear pharmacokinetics of DA-6886 was more obviously observed after oral administration, it appears that the saturation of pre-systemic intestinal and/or hepatic first-pass extraction of DA-6886 at high doses occurred.

## 1. Introduction

Serotonin (5-hydroxytrypamine (5-HT)) released from enterochromaffin cells in the gut mucosa regulates gastrointestinal (GI) motility, the tone of smooth muscles, and mucosal secretion. Among various 5-HT receptors, the key receptors controlling GI functions and sensations are the 5-HT_3_ receptors that produce fast neuronal excitatory responses and 5-HT_4_ receptors that produce prolonged excitatory responses [1]. The 5-HT_4_ receptor agonists have been considered as effective prokinetic drugs for the treatment of GI disorders, such as gastroparesis, chronic constipation, functional dyspepsia, and irritable bowel syndrome (IBS) [2]. The selectivity of 5-HT_4_ receptor agonists is an important determinant of their safety and ultimately the success of their clinical trials. Nonselective 5-HT_4_ receptor agonists, including tegaserod, have reported cardiovascular adverse events [3]. In contrast, highly selective 5-HT_4_ agonists, such as prucalopride, velusetrag, and naronapride, have demonstrated favorable safety and efficacy in the treatment of chronic constipation [4,5]. Prucalopride is the first approved selective 5-HT_4_ receptor agonist, but it has a lower efficacy compared with tegaserod in many preclinical models [6,7]. Other selective 5-HT_4_ receptor agonists, including velusetrag and naronapride, are still in the stage of development [8].

**Figure 1:**
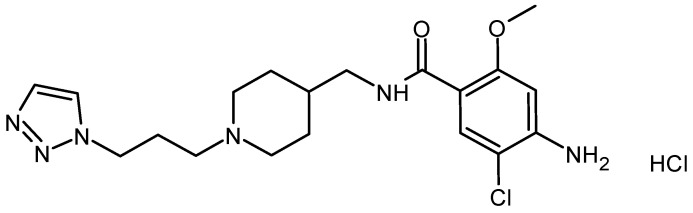
Chemical structure of DA-6886.

DA-6886 (4-amino-5-chloro-2-methoxy-N-[[1-[3-(triazol-1-yl)propyl]piperidin-4-yl]methyl]benzamide hydrochloride, Figure 1) is a novel 5-HT_4_ receptor agonist currently under clinical trial for the potential treatment of constipation-predominant IBS. It showed high affinity and selectivity to human 5-HT_4_ receptor splice variants and significantly improved colonic transit delay in the loperamide-induced constipation mouse model [9]. In addition, the in vivo efficacy of reducing the colonic transit time of oral DA-6886 (5 mg/kg) was similar to that of prucalopride in guinea pigs, showing a human-like distribution of colonic 5-HT_4_ receptors [10]. The affinity of DA-6886 for the human 5-HT_4_ receptor splice variants (5-HT_4a_, 5-HT_4b_, and 5-HT_4d_), which are mainly expressed in the digestive tract, were higher (almost four times) than those of prucalopride, which is the first approved selective 5-HT_4_ receptor agonist [9]. The phase I clinical trials of DA-6886 conducted in South Korea demonstrated that DA-6886 is safe and well tolerated when administered in single and repeated doses to healthy subjects [11].

The aim of this study was to validate the quantitative analysis method of DA-6886 in rat plasma and to evaluate the pharmacokinetics and tissue distribution of DA-6886 in rats. The pharmacokinetics of DA-6886 and its dose-dependency are important data for the design of clinical studies and a comprehensive understanding of safety and efficacy of the drug during preclinical development. In the present study, a liquid chromatography–tandem mass spectrometry (LC–MS/MS) method was developed and validated for the precise and accurate quantification of DA-6886 in rat plasma. The dose-dependency in the pharmacokinetics of DA-6886 was verified following a single intravenous and oral administration of DA-6886 at various doses to rats. The distribution of DA-6886 to various tissues was also studied following its single intravenous administration.

## 2. Materials and Methods

### 2.1. Chemicals and Reagents

DA-6886 (hydrochloride salt form, purity 99.3%, lot no. R10004) was a product of Dong-A ST (Yongin, Korea). Verapamil hydrochloride (internal standard for the analysis of DA-6886, cat no. V4629) and acetic acid (cat no. 40512) were purchased from Sigma-Aldrich (St. Louis, MO, USA). Other reagents and solvents were of reagent or HPLC grade.

### 2.2. Animals

The protocols of animal studies were approved by the Institutional Animal Care and Use Committee (IACUC) of Dong-A Research Center (No. I-1008006, I-1008036, I-1008037, I-1103013) and conducted according to the IACUC guidelines. Male Sprague-Dawley rats purchased from Orient Bio (Sungnam, Korea) were acclimated for 1 week and used for studies at ~8 weeks old. The conditions and procedures used for the housing and handling of rats are similar to those described in other studies [12,13]. The animals were fasted for 18 h and had free access to water before dosing.

### 2.3. Intravenous and Oral Adminitration

The jugular vein (for intravenous administration only) and carotid artery (for serial blood sampling) were cannulated with polyethylene tubing (PE 60) according to reported methods [12,13]. DA-6886 (dissolved in water for injection) at a dose of 2, 10, and 20 mg as free form per kilogram was administered intravenously (1-min infusion) or orally (using gastric gavage) to rats (*n* = 4 each). Blood samples (~250 μL) were collected via the carotid artery at 0, 1 (only for intravenous administration), 5, 15, 30, 60, 120, 180, 300, 420, and 1440 min following the drug administration. The cannula was flushed following each blood sampling to avoid blood clotting using 0.4 mL heparinized (20 units/mL) normal saline. The collected sample was immediately centrifuged and a 100 μL plasma sample was stored at −20 °C until it was used for the LC–MS/MS analyses of DA-6886. 

After the intravenous administration of 10 mg/kg DA-6886 in the same way as above, at 0.5 and 5 h, plasma and major organs (brain, heart, lung, liver, kidney, stomach, small intestine, large intestine, fat, and muscle) were enucleated (*n* = 5 for each time point). Each organ was homogenized by adding five times the volume of sterile distilled water. After centrifugation at 4 °C for 3 min, 20 μL of each supernatant was dispensed and stored frozen at −20 °C.

### 2.4. LC–MS/MS Analysis of DA-6886

The LC–MS/MS system consisted of a Shiseido Nanospace SI-2 LC System (Shiseido, Tokyo, Japan) and an API 4000 QTrap mass spectrometer (AB Sciex, Framingham, MA, USA). The instrument was controlled using the Analyst software (Version 1.5, AB Sciex). The LC–MS/MS method for the determination of DA-6886 concentrations in the samples was developed and validated for its linearity, sensitivity, specificity, precision, accuracy, matrix effect, recovery, stability, and dilution effect [14]. Chromatographic separation was conducted using a reversed-phase HPLC column (Atlantis C18 column; 2.1 × 100 mm; particle size 3 μm). The isocratic elution of the mobile phase (10 mM ammonium acetate in water (pH 5.5) and acetonitrile at a ratio of 40:60 (*v*/*v*)) was accomplished with flow rate of 0.2 mL/min. The column and autosampler temperatures were set at 30 °C and 4 °C, respectively. The total run time was 5 min. 

The eluent was monitored using a multiple reaction monitoring mode of mass spectrometer equipped with an ESI source in positive ion mode. The instrument parameters were as follows: nebulizer gas (50); curtain gas (20); auxiliary gas (50); collision gas (medium); ion spray voltage (5.5 kV); entrance potential (10); and temperature (500 °C). The declustering potential, collision energy, and collision cell exit potential were set at 86, 41, and 10 V for DA-6886 and 101, 37, and 8 V for the IS (verapamil). The precursor-to-product ion transitions applied for quantification were *m*/*z* 407.195 → 184.000 for DA-6886 ([M+H]^+^) and *m*/*z* 455.200 → 165.000 for the IS ([M+H]^+^).

A 300 μL aliquot of IS working solution in acetonitrile (verapamil 50 ng/mL) was added to each 100 μL plasma sample and mixed by vortex to precipitate the protein. A 500 μL of the IS working solution was added to the tissue samples dispensed by 20 μL, respectively. The preparation of calibration standards for tissue samples is detailed in Table S1. After centrifugation (16,000× g, 10 min), the supernatant was transferred to a 96-well plate and a 10 μL aliquot was injected into the LC–MS/MS system for analyses.

### 2.5. Pharmacokinetic Analysis

Pharmacokinetic parameters, such as time-averaged total body clearance (CL) and steady-state volume of distribution (*V*_ss_), were calculated using non-compartmental analyses (WinNonlin ver. 6.1; Pharsight Corporation, Mountain View, CA, USA) [15]. The trapezoidal rule-extrapolation method was used to calculate the total area under the plasma concentration–time curve from time zero to infinity (AUC_0–__∞_) [16]. The maximum plasma concentration (*C*_max_) and time to reach *C*_max_ (*T*_max_) were determined from the observed data. The extent of absolute oral bioavailability (*F*) was estimated by comparing the AUC_0–__∞_ of DA-6886 after oral administration with the AUC_0–__∞_ of DA-6886 after the intravenous administration of the same dose. All data are presented as mean ± standard deviation (S.D.), except median (range) for *T*_max_. An assessment of the dose proportionality was performed using the reported power model based on 90% confidence interval (CI) for the ratio of dose normalized means (R_dnm_) lying within pre-specified acceptance limits (0.8–1.25) [17,18].

### 2.6. Statistical Analysis

GraphPad Prism (ver. 7.05, GraphPad Software, Inc., San Diego, CA, USA) was utilized for statistical analysis. To compare three means of unpaired data, the Kruskal–Wallis test and a post hoc Dunn’s multiple comparison test were applied. Statistical significance was concluded when a *p* value of <0.05 was obtained. 

## 3. Results

### 3.1. Analytical Method Validation

#### 3.1.1. Linearity, LLOQ, and Specificity

The calibration curve was constructed by calculating the peak area ratio of DA-6886 to the peak area of verapamil (IS), and it showed a linearity of r = 0.9980 to 0.9997 in the concentration range of 2 to 2000 ng/mL. The relative error (RE) for the concentration of each standard sample, which was inversely converted using the calibration curve of DA-6886, was −3.14 to 3.04%, and the coefficient of variation (CV) was 1.73 to 5.21%. The lowest limit of quantitation (LLOQ) of this assay was that the peak area of DA-6886 was at least five times higher than that of the empty plasma sample, and the concentration was determined to be less than 20% error in precision and accuracy. The LLOQ of this assay was determined to be 2 ng/mL (Figure S1), and at this concentration, the CV value of DA-6886 was 5.74% and the RE value was −6.80% (Table 1). To evaluate the specificity of this assay method, blank plasma obtained from six rats and LLOQ samples were analyzed, and the area of the interference peak corresponding to retention time of the analyte in blank samples and the average area of the LLOQ sample peak were compared. In the case of blank samples, a disturbance peak of 0–0.164% of the LLOQ peak was observed for each individual, and all criteria were satisfied within 20% [14]. 

#### 3.1.2. Precision and Accuracy

The precision of the assay is determined by the CV for the concentration of DA-6886 measured using QC samples at low (6 ng/mL), medium (100 ng/mL), and high (1600 ng/mL) concentrations. The CV value for intra-day precision (*n* = 5) for each concentration sample was 2.16–5.00% (Table 1), and the CV value for inter-day precision for 5 days (*n* = 5) was 2.45~4.12% (Table 1). These results of intra-day precision and day-to-day precision met the acceptance criteria within 15% [14]. The accuracy of the analytical method is based on the RE for the concentration of DA-6886 measured using QC samples at low (6 ng/mL), medium (100 ng/mL), and high (1600 ng/mL) concentrations. The RE value of DA-6886 for intra-day accuracy (*n* = 5) for each concentration sample was −3.25 to 2.58% (Table 1), and the RE value of DA-6886 for inter-day accuracy for 5 days (*n* = 5) was 0.733~3.33% (Table 1). The results of intra-day accuracy and day-to-day accuracy were acceptable (within ±15%) [14].

#### 3.1.3. Matrix Effect and Recovery

After the pretreatment of the rat plasma, the matrix effect of the biological sample was evaluated by adding a working standard of DA-6886 equivalent to the low concentration QC (LQC) and high concentration QC (HQC) samples. The CV of the peak area for each concentration were 5.83% (at 6 ng/mL) and 3.06% (at 1600 ng/mL), which satisfies 15% or less. The recovery of DA-6886 during the sample preparation was estimated by comparing the peak area of DA-6886 in the LQC and HQC samples (extracted samples) to that of the unextracted samples prepared by adding the working standard of DA-6886 equivalent to the LQC (6 ng/mL) and HQC (1600 ng/mL) after the pretreatment of the empty plasma. The recovery of DA-6886 was 102% and 88.1% in LQC and HQC, respectively, and the CV value was 1.69~6.34%, which satisfies the criteria of less than 15% [14].

#### 3.1.4. Stability of the DA-6886 and Dilution Effect

The mean of the measured values for LQC (6 ng/mL) and HQC (1600 ng/mL) following storage with various conditions (room temperature for 4 h, −20 °C for 14 days, post-preparation 4 °C for 24 h, three freeze–thaw cycles) were compared with the mean measured values of the immediately prepared QC sample (Table S2). The CV value of samples following various storage conditions were below 9.48%, which satisfies 15% or less, and the relative concentration value satisfies the standard of 85~115%. These results demonstrated that DA-6886 was stable during the storage, preparation, and analysis of samples. In order to secure the quantitation for samples exceeding 2000 ng/mL, the highest quantitation limit of this assay, rat plasma samples (*n* = 3) with a concentration of DA-6886 of 40,000 ng/mL were prepared and measured by pre-treatment after diluting them 20-fold with empty plasma. The CV value for the measured concentration of DA-6886 diluted 20 times was 4.13%, and the RE value was −4.00%. 

### 3.2. Pharmacokinetics of DA-6886 following Intravenous Administration

**Figure 2:**
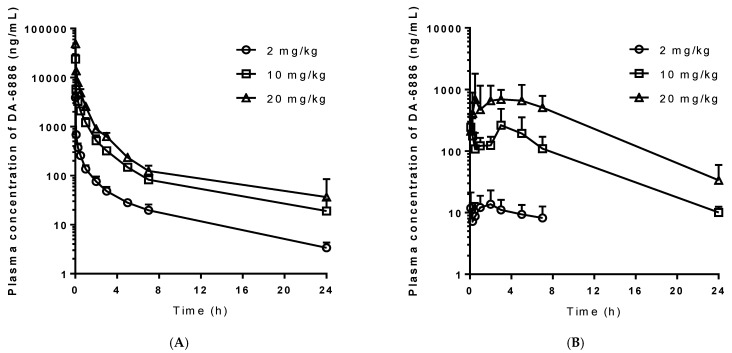
Mean arterial plasma concentration–time profiles of DA-6886 following intravenous (**A**) and oral (**B**) administration of DA-6886 at doses of 2, 10, and 20 mg/kg to rats (*n* = 4 each). Data are expressed as mean ± S.D.

The above-validated LC–MS/MS method was applied to the pharmacokinetic studies of DA-6886 in rats. After a single intravenous administration of various doses of DA-6886 (2, 10, and 20 mg/kg) to rats, the mean plasma concentrations of DA-6886 versus time are plotted in Figure 2A, and the relevant pharmacokinetic parameters are listed in Table 2. The significantly faster CL in the low dose (2 mg/kg) group compared with the high dose (20 mg/kg) group resulted in significantly smaller dose-normalized AUC_0–∞_ in the low dose group.

### 3.3. Pharmacokinetics of DA-6886 following Oral Administration

Following a single oral administration of DA-6886 (2, 10, and 20 mg/kg) to rats, the mean plasma concentration of DA-6886 versus time profiles are presented in Figure 2B, and the relevant pharmacokinetic parameters are listed in Table 3. Similar to the intravenous study, the dose-normalized AUC_0–∞_ in the low dose group was significantly smaller than in the high dose group. Greater dose-normalized AUC_0–∞_ and *C*_max_ were observed with an increasing oral dose of DA-6886. As a result, the *F* value showed a tendency to increase with an increasing dose. 

### 3.4. Assessment of Dose Proportionality of DA-6886

Following a single administration of DA-6886 (2, 10, and 20 mg/kg) to rats, the slope of the log-transformed AUC and/or *C*_max_ values versus log dose and their 90% CI were estimated based on the power model (Table 4). The ratio of dose-normalized geometric mean and its 90% CI of AUC and *C*_max_ were also summarized in Table 4. The maximal proportional dose ratio indicates that the proportionality of AUC can be concluded within ~2-fold intravenous dose ratio. 

### 3.5. Tissue Distribution of DA-6886

**Figure 3:**
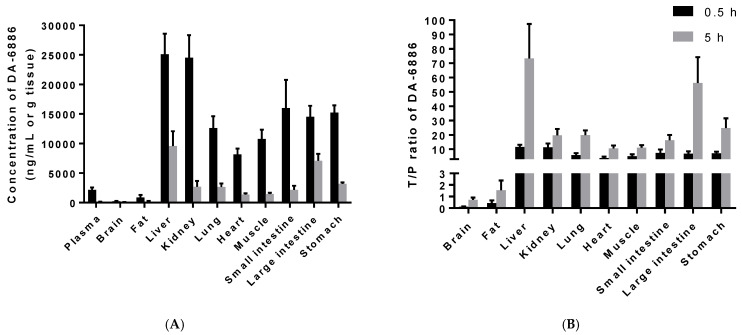
Tissue concentration (**A**) and T/P (Tissue (ng/g tissue) to plasma (ng/mL)) ratio (**B**) of DA-6886 at 0.5 h (black bar, *n* = 5) and 5 h (gray bar, *n* = 5) after single intravenous administration of DA-6886 hydrochloride at a dose of 10 mg/kg to male Sprague-Dawley rats. Data are expressed as mean ± S.D.

After a single intravenous administration of 10 mg/kg DA-6886, the concentration and tissue-to-plasma concentration ratio (T/P ratio) in each tissue after 0.5 h and 5 h are shown in Figure 3. DA-6886 was observed at a higher concentration than plasma with a T/P ratio of greater than 1 in all organs except the brain (0.5 and 5 h) and fat (0.5 h).

## 4. Discussion

The LC–MS/MS method developed for the quantification of DA-6886, a novel 5-HT_4_ receptor agonist for the treatment of IBS, in rat plasma showed acceptable performances in specificity, linearity, sensitivity, precision, and accuracy of analysis. DA-6886 demonstrated robust recovery and stability in rat plasma during storage, sample preparation, and analysis. The successfully validated method was applied to evaluate the pharmacokinetics of DA-6886 in rats at various dose levels. 

Linear pharmacokinetics with dose-proportional exposure allows us to easily assess dose–effect and dose–toxicity relationships. On the other hand, nonlinear pharmacokinetics may make it difficult to predict effective doses in clinical trials. Therefore, when nonlinear pharmacokinetic parameters are observed in preclinical studies, it is important to elucidate the underlying mechanisms and to quantitively predict nonlinearities in humans [19,20]. The preclinical pharmacokinetic study of DA-6886 in rats was designed to assess not only the pharmacokinetic parameters of DA-6886 in rats, but also the dose-dependency in pharmacokinetic parameters and the dose–exposure relationship. Following the intravenous administration of DA-6886 at doses of 2, 10, and 20 mg/kg, the CL in the low-dose group (37.7 mL/min/kg) was significantly faster than that in the high-dose group (26.0 mL/min/kg). As a result, the dose-normalized AUC of DA-6886 tended to increase with an increasing intravenous dose. The slope of the log-transformed AUC versus the log intravenous dose was slightly greater than one (1.066), and the 90% CI of R_dnm_ of AUC did not lie within the acceptance interval (0.8, 1.25) [17]. Based on the maximal proportional dose ratio (1.96; Table 4) [17], the AUC of DA-6886 would be dose-proportional within the dose range of a double apart. The *V*_ss_ of DA-6686 showed similar values irrespective of the intravenous dose. The *V*_ss_ of DA-6886 was approximately 10-fold larger than the reported total body water in rats (0.668 L/kg) [21], suggesting that a significant amount of the drug is distributed into the tissues. This is also consistent with the high distribution of DA-6886 to tissues other than the brain and fat. DA-6886 showed particularly high distribution to the liver, kidney, lung, and digestive tract (Figure 3). The high distribution of DA-6886 to the target organ, the digestive tract, suggests that it has adequate efficacy. Prucalopride, the first approved selective 5-HT_4_ receptor agonist, also showed high distribution in the digestive tract in rats following oral administration [22]. On the other hand, the low distribution of DA-6886 to the brain tissue suggests that DA-6886 cannot pass through the blood–brain barrier and the potential for adverse effects on the central nervous system is low.

The dose-normalized AUC of DA-6886 also increased with an increasing oral dose. The slope of the log-transformed AUC and *C*_max_ values versus the log oral dose (1.858 and 1.875, respectively) were much greater than one and the respective slope of intravenous AUC (1.066). Moreover, the 90% CI of R_dnm_ of oral AUC values lay completely outside of the acceptance interval (0.8, 1.25). Therefore, when DA-6886 was administered orally, the non-proportional increase in AUC according to the dose increase was more pronounced than when administered intravenously. As a result, the *F* value also increased with an increasing oral dose (18.9% to 55.0%), which is considered to be due to the saturation of the intestinal and/or hepatic first-pass extraction during the absorption process in high dose. This nonlinear pharmacokinetics of DA-6886 observed in rats suggests attention is warranted when carrying out dose escalation during phase I clinical trials. It should be noted that the dose-dependency observed in preclinical pharmacokinetic studies may not necessarily be to the same extent in humans. For example, a drug with different metabolic pathways between species, such as the metabolism in rats with a low Michaelis constant and the metabolism in humans with a high capacity, exhibited nonlinear pharmacokinetics in rats and linear pharmacokinetics in humans [20]. Based on an internal report, dealkylation was the major metabolic pathway of DA-6886 in both rats and humans. Furthermore, as a result of experiments using selective inhibitors and recombinant human cytochrome P450s (CYPs), it was confirmed that the dealkylation of DA-6886 is mediated by CYP3A4, and that DA-6886 is a reversible inhibitor of CYP3A4 (IC50 value of ~30 μM) in human liver microsomes. 

In addition to the saturation of the first-pass metabolism of DA-6886, the nonlinear (saturable) distribution of DA-6886 into the intestine during the absorption process could be attributed in part to the dose-dependent pharmacokinetics of DA-6886 after oral administration. Unlike other 5-HT receptors, 5-HT_4_ receptors are distributed in enterocytes as well as enteric neurons and smooth muscles [2]. Therefore, DA-6886 is expected to be distributed in a significant amount to enterocytes and other parts of the intestinal tissue. In fact, it was confirmed that the distribution of DA-6886 into the digestive tract was significant even after intravenous administration. 

In conclusion, DA-6886 demonstrated nonlinear pharmacokinetics after oral administration (2–20 mg/kg) and showed a larger *F* value with an increasing oral dose. The saturation of processes during absorption, such as first-pass metabolism and/or distribution to the intestinal tissue, could be the possible cause of dose-dependency of DA-6886 after oral administration. To quantitatively predict the nonlinearity of the pharmacokinetics of DA-6886 in humans, further studies on the enzyme kinetics of the hepatic and intestinal metabolism in rats and humans are needed. The validated quantification method and the results of this pharmacokinetic study in rats can be utilized for further pharmacokinetic studies of DA-6886. The tissue distribution results can also be applied to the future development of physiologically based pharmacokinetic models of DA-6886.

## Figures and Tables

**Table 1:** Precision and accuracy of the analytical method for the determination of DA-6886 in rat plasma.

Nominal Concentration (ng/mL)	Intra-Day (*n* = 5)			Inter-Day (*n* = 5)		
	Measured Concentration (Mean, ng/mL)	Precision (CV, %)	Accuracy (RE, %)	Measured Concentration (Mean, ng/mL)	Precision (CV, %)	Accuracy (RE, %)
2 (LLOQ)	1.86	5.74	−6.80	-	-	-
6 (LQC)	6.14	5.00	2.27	6.04	2.45	0.733
100	103	2.16	2.58	102	4.05	2.40
1600 (HQC)	1550	3.14	−3.25	1650	4.12	3.33

**Table 2:** Pharmacokinetic parameters (mean ± S.D.) for DA-6886 after single intravenous administration at doses of 2, 10, and 20 mg/kg to rats.

Parameters	2 mg/kg (*n* = 4)	10 mg/kg (*n* = 4)	20 mg/kg (*n* = 4)
AUC_0–∞_ (ng∙h/mL) ^1,^ ^2^	908 ± 153	6360 ± 1130	13,000 ± 1910
Terminal half-life (h)	5.20 ± 2.04	7.00 ± 2.70	6.54 ± 3.83
CL (mL/min/kg) ^2^	37.7 ± 7.60	26.8 ± 4.51	26.0 ± 3.37
*V*_ss_ (L/kg)	7.84 ± 2.21	5.64 ± 1.49	4.91 ± 3.14

^1^ Dose-normalized (based on 1 mg/kg) values were compared for statistical analysis. ^2^ 2 mg/kg was significantly different (*p* < 0.05) from 20 mg/kg.

**Table 3:** Pharmacokinetic parameters (mean ± S.D.) for DA-6886 after single oral administration at doses of 2, 10, and 20 mg/kg to rats.

Parameters	2 mg/kg (*n* = 4)	10 mg/kg (*n* = 4)	20 mg/kg (*n* = 4)
AUC_0–∞_ (ng∙h/mL) ^1, 2^	171 ± 153	1960 ± 819	7160 ± 2150
Terminal half-life (h)	7.51 ± 3.95	5.93 ± 2.94	4.64 ± 1.42
*C*_max_ (ng/mL) ^1, 2^	17.0 ± 6.80	393 ± 131	1440 ± 653
*T*_max_ (h) ^3^	1.5 (0.5–3)	1.63 (0.0833–5)	4 (0.5–7)
*F* (%)	18.9	30.9	55.0

^1^ Dose-normalized (based on 1 mg/kg) values were compared for statistical analysis. ^2^ 2 mg/kg was significantly different (*p* < 0.05) from 20 mg/kg. ^3^ Median (range).

**Table 4:** Dose proportionality assessment of DA-6886 after single intravenous and oral administration at doses of 2, 10, and 20 mg/kg to rats.

Parameters	Slope of the Log-Transformed Parameter Versus Log Dose, 90% CI	Ratio of Dose-Normalized Geometric Mean (Rdnm), 90% CI	Dose Proportionality	Maximal Proportional Dose Ratio ^3^	Threshold Dose Ratio to Reject Proportionality ^3^
AUC_0–∞_ after intravenous administration	1.066 (0.8513–1.332)	1.164 (0.7101–2.148)	Inconclusive ^1^	1.96	-
AUC_0–∞_ after oral administration	1.858 (1.171–3.124)	7.211 (1.483–133.0)	Not proportional ^2^	1.11	3.68
*C*_max_ after oral administration	1.875 (0.9649–4.528)	7.499 (0.9224–3373)	Inconclusive ^1^	1.07	-

^1^ Proportionality was inconclusive because the 90% CI for R_dnm_ contains 1. ^2^ Proportionality was rejected because the 90% CI for R_dnm_ was completely outside of acceptance range (0.8, 1.25). ^3^ These values were calculated according to the reference [17].

## Data Availability

The main part of the research data is contained in the article and Supplementary Materials. Other data including chromatograms are available from the corresponding author upon request.

## Supplementary Materials

The following supporting information can be downloaded at: https://www.mdpi.com/article/10.3390/pharmaceutics14040702/s1, Table S1: Preparation of calibration standards for tissue homogenates, Figure S1: Representative MRM chromatograms of DA-6886 (I) and the IS verapamil (II) in rat plasma sample, Table S2: Stability of DA-6886 in rat plasma at different storage conditions.
