# Supplementary material for: Pharmacokinetics of DA-6886, A New 5-HT4 Receptor Agonist, in Rats
Source: Pharmaceutics. 2022 Mar 25;14(4):702. doi: 10.3390/pharmaceutics14040702 (PMC9024849; doi:10.3390/pharmaceutics14040702)
Supplement: Supplementary file 1 [file pharmaceutics-14-00702-s001.zip › pharmaceutics-1629108-SM.pdf]

pharmaceutics

Supplementary Materials: Pharmacokinetics of DA-6886, A New 5-HT_4 Receptor Agonist, in Rats

Dae Young Lee and Hee Eun Kang

Table S1. Preparation of calibration standards for tissue homogenates.

Table S1(a). Grouping rat tissues based on blood perfusion, muscle mass and other properties.

Group	Tissues	Properties
1	Brain	Contains a large amount of phospholipids.
2	Heart, lungs, liver, kidneys	High blood flow and red tissue.
3	Stomach, small intestine, large intestine, muscle	Digestive system, lots of muscles.
4	Fat	Lots of fat.

Table S1(b). Calibration standards for each group of tissues.

Group	Tissue standards (ng/g tissue)
1, 4	10, 30, 100, 300, 1000, 3000
2, 3	100, 300, 1000, 3000, 10000, 30000

(a)

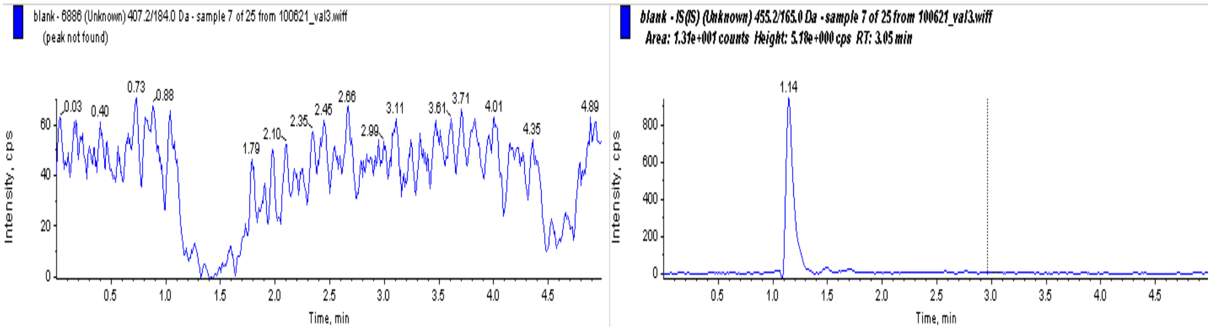

(I) **(II)**

(b)

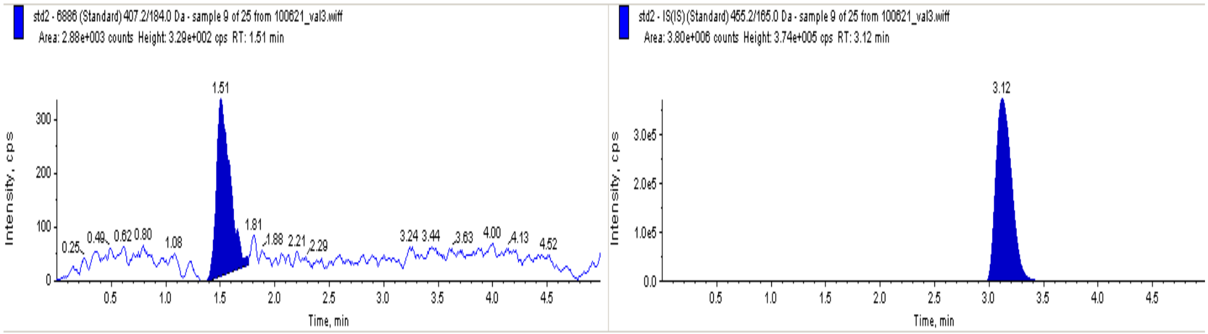

(I) **(II)**

Figure S1. Representative MRM chromatograms of DA-6886 (I) and the IS verapamil (II) in rat plasma sample: (a) blank plasma; (b) blank plasma spiked with LLOQ level of DA-6886 (2 ng/mL) or mixed with the IS at working concentrations (50 ng/mL).

Table S2. Stability of DA-6886 in rat plasma at different storage conditions (n = 3).

Storage conditions	QC concentration (ng/mL)	Initially measured concentration (ng/mL)		Finally measured concentration (ng/mL)		
		Mean ± S.D.	CV (%)	Mean ± S.D.	CV (%)	Relative Concentration (%)
**Short term**[1]	6 (LQC)	6.12 ± 0.400	6.54	6.38 ± 0.403	6.31	104
	1600 (HQC)	1563 ± 20.8	1.33	1733 ± 40.4	2.33	111
Post-preparative[2]	6 (LQC)	6.12 ± 0.400	6.54	6.05 ± 0.574	9.48	99.0
	1600 (HQC)	1563 ± 20.8	1.33	1523 ± 60.3	3.96	97.4
Long term storage & Freeze-thaw[3]	6 (LQC)	6.12 ± 0.400	6.54	5.97 ± 0.166	2.78	97.5
	1600 (HQC)	1563 ± 20.8	1.33	1767 ± 15.3	0.865	113

[1] Storage at room temperature for 4 h; [2] Storage of processed samples at 4°C for 24 h; [3] Storage at –20°C over 14 days and three cycles of –20°C to room temperature.
